# Misidentification of meticillin-resistant *Staphylococcus aureus* by the Cepheid Xpert MRSA NxG assay, the Netherlands, February to March 2021

**DOI:** 10.2807/1560-7917.ES.2021.26.37.2100800

**Published:** 2021-09-16

**Authors:** Artur J Sabat, Erik Bathoorn, Monika A Chlebowicz-Fliss, Viktoria Akkerboom, Inge Kamphuis, Claudy Oliveira dos Santos, Alexander W Friedrich

**Affiliations:** 1 University of Groningen, University Medical Center Groningen, Department of Medical Microbiology, Groningen, the Netherlands; 2 Isala Hospital, Laboratory for Medical Microbiology and Infectious Diseases, Zwolle, the Netherlands

**Keywords:** *Staphylococcus aureus*, SCC*mec*, composite island, real-time PCR, Cepheid

## Abstract

We describe two false-negative results in the detection of meticillin-resistant *Staphylococcus aureus* (MRSA) of sequence type 398 and *spa* type t011 using the Cepheid Xpert MRSA NxG assay. The isolates were recovered in late February and early March 2021 from two patients in different hospitals in the northern Netherlands. Variations between the two isolate genomes indicate that this MRSA strain might have been spreading for some time and could have disseminated to other regions of the Netherlands and other European countries.

In this report, we describe two instances of false-negative results in the detection of meticillin-resistant *Staphylococcus aureus* (MRSA) using the Cepheid Xpert MRSA NxG assay (Sunnyvale, California, United States (US)). The objective of this study was to elucidate a reason for the assay failure by applying whole genome sequencing. Moreover, the genome sequence data were used to assess the similarity between the study isolates.

## Isolate identification

The first MRSA isolate (designated NL1) was obtained from a nasal sample of an asymptomatic veal farmer in his late 40s in February 2021, as a part of MRSA screening at a hospital in Zwolle, the Netherlands. The second MRSA isolate (designated UMCG578) was recovered from a sinus pus sample from a male patient in his early 70s with chronic purulent sinusitis at the beginning of March 2021 in a hospital in Groningen, the Netherlands. The two Dutch hospitals are located 104 km apart. 

The isolates were identified as *S. aureus* by matrix-assisted laser desorption/ionization–time of flight mass spectrometry (MALDI-TOF MS) (Bruker Daltonics, Billerica, Massachusetts, US). In both cases, the Xpert MRSA NxG assay was performed on pure colonies cultured and taken for testing from blood agar plates. The Xpert MRSA NxG assay detected the *mecA* target but not chromosome-SCC*mec* junction in both MRSA isolates.

## Antibiotic resistance testing and results

Antibiotic resistance properties of isolates were further characterised. The minimum inhibitory concentration (MIC) values of 22 antibiotics were determined by Etest (bioMérieux, Marcy-l’Étoile, France) and the results were interpreted according to the European Committee on Antimicrobial Susceptibility Testing (EUCAST) guidelines [1]. Isolates NL1 and UMCG578 were phenotypically resistant to beta-lactams (benzylpenicillin, oxacillin and cefoxitin) but susceptible to ceftaroline (Table 1). The isolates were also resistant to sulfamethoxazole/trimethoprim, gentamicin, kanamycin, tobramycin and tetracycline. Moreover, isolate NL1 was resistant to clindamycin and erythromycin.

**Table 1:** Characterisation of antibiotic resistance of meticillin-resistant *Staphylococcus aureus* isolates, the Netherlands, February–March 2021 (n = 2)

**Antibiotic**	**Isolate**					
	**NL1**			**UMCG578**		
	**R/S**	**MIC** **(µg/mL)**	**Acquired resistance gene**	**R/S**	**MIC** **(µg/mL)**	**Acquired resistance gene**
Benzylpenicillin	R	24	*blaZ*	R	24	*blaZ*
Oxacillin	R	> 256	*mecA*	R	> 256	*mecA*
Cefoxitin	R	192	*mecA*	R	96	*mecA*
Ceftaroline	S	1	NF	S	0.75	NF
Vancomycin	S	1.5	NF	S	1	NF
Teicoplanin	S	1	NF	S	1	NF
Clindamycin	R	> 256	*erm(T)*	S	0.125	NF
Linezolid	S	2	NF	S	1	NF
Rifampicin	S	0.012	NF	S	0.008	NF
Sulfamethoxazole/trimethoprim	R	> 32	*dfrK*	R	> 32	*dfrK*
Gentamicin	R	16	*aac(6')-aph(2”)*	R	8	*aac(6')-aph(2”)*
Kanamycin	R	256	*aac(6')-aph(2”)*	R	256	*aac(6')-aph(2”)*
Tobramycin	R	32	*aac(6')-aph(2”)*	R	4	*aac(6')-aph(2”)*
Ciprofloxacin	S	0.25	NF	S	0.25	NF
Erythromycin	R	> 256	*erm(T)*	S	0.25	NF
Mupirocin	S	0.25	NF	S	0.125	NF
Tetracycline	R	> 256	*tet(L), tet(M)*	R	> 256	*tet(M)*
Chloramphenicol	S	8	NF	S	8	NF
Daptomycin	S	0.19	NF	S	0.25	NF
Fusidic acid	S	0.25	NF	S	0.25	NF
Amikacin	S	2	*aac(6')-aph(2”)*	S	2	*aac(6')-aph(2”)*
Moxifloxacin	S	0.047	NF	S	0.047	NF

MIC: minimum inhibitory concentration; NF: not found; R: resistant; S: susceptible.

## Whole genome sequencing

The cells were lysed using the lysostaphin/lysozyme enzyme and total DNA was purified using the MagAttract HMW DNA Kit (Qiagen, Hilden, Germany). DNA was quantified using a Qubit 2.0 fluorometer (ThermoFisher Scientific, Waltham, Massachusetts, US) and the quality was assessed by the 2200 TapeStation software (Agilent Technologies, Santa Clara, California, US). A NanoDrop 2000c spectrophotometer (ThermoFisher Scientific) was used to measure the purity of extracted DNA. 

To obtain the complete genome sequences for each isolate, Illumina genomic libraries were prepared using a Nextera XT kit (Illumina, San Diego, California, US) and sequenced on a MiSeq platform (Illumina) with a 2 × 300 bp paired-end protocol. Oxford Nanopore sequencing libraries were prepared using the Ligation Sequencing Kit (SQK-LSK109), and sequencing was carried out on a MinION device using flow cell type R9.4.1 (FLO-MIN106D, Nanopore, Oxford, United Kingdom). Nanopore reads were de novo assembled with SeqMan NGen assembler version 17.2.1.61 (DNASTAR, Madison, Wisconsin, US). Chromosomes were obtained as single contigs with a depth of coverage of 169.65 × for NL1 and 125.48 × for UMCG578. Chromosome consensus sequences were refined using the Illumina reads and the SeqMan NGen assembler with automated polishing workflow. Polished assemblies were manually corrected using SeqMan Pro (DNASTAR). The remaining Illumina reads, which were not mapped to the chromosomes during refinement step, were de novo assembled using SeqMan NGen. The resulting contigs were used in Basic Local Alignment Search Tool (BLASTn) against GenBank to identify plasmids. Automated genome annotation was performed using the NCBI Prokaryotic Genome Annotation Pipeline (https://www.ncbi.nlm.nih.gov/genome/annotation_prok).

### Nucleotide sequence accession numbers

The sequences of the chromosome of *S. aureus* NL1 and its three plasmids (pNL1–01, pNL1–02 and pNL1–03) have been deposited in GenBank under accession numbers CP077741–CP077744. The sequences of the chromosome of *S. aureus* UMCG578 and its two plasmids (pUMCG578–01 and pUMCG578–02) have been deposited in GenBank under accession numbers CP077738–CP077740.

### Molecular characterisation and single nucleotide polymorphism analysis

**Figure 1:**
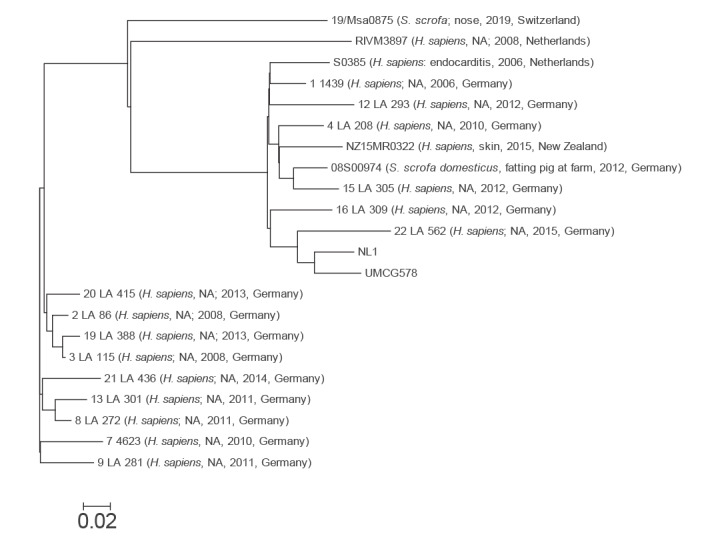
Phylogenetic relationships of *Staphylococcus aureus* sequence type (ST) 398 isolates, the Netherlands, February–March 2021 (n = 22)

NA: not available; SNP: single nucleotide polymorphism.

The complete chromosome sequence of isolate NL1 consisted of 2,911,340 bp and UMCG578 had 2,871,141 bp. Based on in silico analysis, the isolates were identified as multilocus sequence type (ST) 398 and *spa* type t011, which is the most frequent lineage of livestock-associated MRSA in the Netherlands [2,3]. The complete chromosome sequences of the two study isolates and 20 other ST398 isolates were uploaded to the CSI Phylogeny 1.4 server (https://cge.cbs.dtu.dk/services/CSIPhylogeny) in order to investigate their single nucleotide polymorphism (SNP)-based phylogeny. The selection of the ST398 isolates was obtained based on the BLASTn analysis. Of all sequences deposited in the GenBank database, the selected 20 ST398 chromosomes were most genetically related to the chromosome of the NL1 isolate. Genome-wide SNP results revealed that the NL1 chromosome sequence was the most related to that of UMCG578 (Figure 1), although the two sequences differed by 59 SNPs.

Phylogenetic maximum likelihood tree constructed on the basis of SNPs was obtained by CSI phylogeny 1.4 (https://cge.cbs.dtu.dk/services/CSIPhylogeny). Isolate attributes (host; isolation source; collection year; geographic origin). The scale bar indicates the evolutionary distance between the sequences determined by 0.02 substitutions per nucleotide at the variable positions.

The following isolates were analysed: NL1; UMCG578; 1_1439 (GenBank accession number: LT992456); 2_LA_86 (GenBank accession number: LT992463); 3_LA_115 (GenBank accession number: LT992464); 4_LA_208 (GenBank accession number: LT992466); 7_4623 (GenBank accession number: LT992458); 08S00974 (GenBank accession number: CP020019); 8_LA_272 (GenBank accession number: LT992461); 9_LA_281 (GenBank accession number: LT992460); 12_LA_293 (GenBank accession number: LT992468); 13_LA_301 (GenBank accession number: LT992470); 15_LA_305 (GenBank accession number: LT992469); 16_LA_309 (GenBank accession number: LT992467); 19_LA_388 (GenBank accession number: LT992474); 19Msa0875 (GenBank accession number: CP047646); 20_LA_415 (GenBank accession number: LT992475); 21_LA_436 (GenBank accession number: LT992476); 22_LA_562 (GenBank accession number: LT992477); NZ15MR0322 (GenBank accession number: LT699704); RIVM3897 (GenBank accession number: CP013621); S0385 (GenBank accession number: AM990992).

### Description of the staphylococcal cassette chromosome *mec* composite island

**Figure 2:**
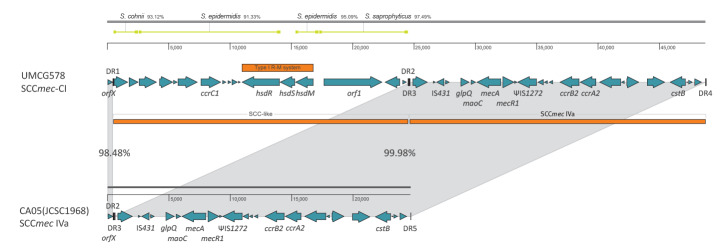
Structural comparison of the staphylococcal cassette chromosome *mec* composite island from *Staphylococcus aureus* isolate UMCG578 with SCC*mec* IVa from *S. aureus* isolate CA05(JCSC1968), the Netherlands, February–March 2021 (n = 2)

CI: composite island; DR: direct repeat sequences; SCC*mec*: staphylococcal cassette chromosome *mec*.

Genome-wide analysis revealed a novel organisation of the SCC*mec* composite island (SCC*mec*-CI) in these two isolates (Figure 2). The nucleotide sequences of SCC*mec*-CI in the isolates were 50,197 bp (NL1) and 48,328 bp (UMCG578). In both isolates, SCC*mec*-CI was composed of the SCC-like region adjacent to the *orfX* gene, followed by the SCC*mec* type IVa region. The SCC-like region, which carries the *ccrC1* gene, was almost identical in both isolates with the exception of a gene encoding putative immunoglobulin (Ig) domain-containing protein. This gene differed in size between the isolates because of 267 nt sequence repeats; NL1 has 15 repeats, while UMCG578 has eight. Therefore, the SCC-like sequence of isolate NL1 was longer (26,033 bp) than that of isolate UMCG578 (24,164 bp). 

The numbers in the rulers are designated relative to the position of A in the ATG start codon of the *orfX* gene. Subregions of the SCC-like region showing the highest similarity to different staphylococcal species are indicated by corresponding line segments (light green). Genes are indicated by horizontal arrows (teal blue). Only the following selected genes are annotated: 23S rRNA methyltransferase RlmH (*orfX*) containing the SCC*mec* insertion site, type I restriction-modification (R-M) system endonuclease subunit R (*hsdR*), type I R-M system endonuclease subunit S (*hsdS*), type I R-M system DNA methylase (*hsdM*), Ig domain-containing protein (*orf1*), glycerophosphoryl diester phosphodiesterase (*glpQ*), MaoC family dehydratase (*maoC*), the determinant encoding resistance to meticillin (*mecA*) and its regulatory gene (*mecR1*), the SCC*mec* cassette recombinases (*ccrC1*, *ccrA2* and *ccrB2*), persulfide dioxygenase-sulfurtransferase (*cstB*) and the transposases of IS*256*, IS*431* and IS*1272*. The vertical bars indicate DRs. Sequences of DRs: DR1 (GAAGCGTACCACAAATAA), DR2 (GAAGCATATCATAAATGA), DR3 (GAAGCTTATCATAAGTAA), DR4 (GAGGCTTATCATAAATGA), and DR5 (GAGGCGTATCATAAGTGA).

Additionally, the SCC-like region displayed a high degree of mosaicism. It was composed of several sub-regions, each of which showed the highest similarity to different staphylococcal species (Figure 2). The SCC*mec* IVa region in both NL1 and UMCG578 was the same size (24,164 bp) and the sequences were almost identical, differing by only a single nucleotide. Moreover, BLAST searches in the GenBank database showed that the SCC*mec* IVa sequences from both isolates were almost identical to that of *S. aureus* isolate CA05 (JCSC1968; identity: 99.98%; GenBank accession number: AB063172) (Figure 2).

### Genetic diversity of the chromosomes

The overall gene content showed some differences in these two isolates (Table 2). Isolate NL1 carried the *Staphylococcus* prophage 96 (size: 42,811 bp; total proteins: 65; position: 1985517.2028327), while this prophage was absent in isolate UMCG578. Both isolates carried the StauST398–2 and StauST398–3 prophages. While the StauST398–3 prophage was identical in both isolates, StauST398–2 differed between isolates by several partial or entire gene deletions. Isolates NL1 and UMCG578 harboured 14 copies of an IS*256*-like insertion sequence per genome. In total, 18 different IS*256*-like insertion loci were identified on the chromosomes of the two isolates, indicating that 10 loci were the same in both isolates and at least four transpositions of the IS*256*-like element had occurred.

**Table 2:** Gene content of the chromosomes of meticillin-resistant *Staphylococcus aureus* isolates, the Netherlands, February–March 2021 (n = 2)

**Features annotated**	**NL1** **(bp)**	**UMCG578** **(bp)**
Genes (total)	2,908	2,819
Coding DNA sequences (total)	2,826	2,734
Coding DNA sequences (with protein)	2,743	2,648
Genes (RNA)	82	85
rRNAs (5S, 16S, 23S)	7, 6, 6	8, 7, 7
tRNAs	59	59
ncRNAs	4	4
Pseudogenes	83	86

rRNA: ribosomal RNA; tRNA: transfer RNA; ncRNA: non-coding RNA.

### Plasmid content

The NL1 and UMCG578 isolates had different plasmid profiles. Both isolates harboured a small cryptic plasmid of 1,373 bp in size (designated pNL1–01 or pUMCG578–01) with an identical nucleotide sequence. This plasmid was highly divergent from any nucleotide sequence deposited in the GenBank database. In isolate NL1, two other plasmids, pNL1–02 and pNL1–03, were also found. Plasmid pNL1–02 had a size of 11,899 bp and carried the *tet(L)* gene (conferring resistance to tetracycline) and *erm(T)* genes (conferring resistance to clindamycin and erythromycin). This plasmid also carried the *cadD* gene encoding cadmium resistance determinant. Plasmid pNL1–02 showed the highest similarity (query cover: 83%; identity: 99.98%) to plasmid pUR2941 from the human isolate of MRSA ST398 [4]. The biggest plasmid of NL1 was designated pNL1–03 and had a size of 17,455 bp. This plasmid shared the highest identity (query cover: 87%; identity: 99.98%) with plasmid 2 from *S. aureus* strain NZ15MR0322. The pNL1–03 plasmid possessed the gene encoding CadD family cadmium resistance transporter and the gene encoding tetronasin resistance protein. The Cad proteins of plasmids pNL1–02 and pNL1–03 were not identical and shared 84% amino acid similarity. The antibiotic compound tetronasin is used as a growth-promotant in animal farms with activity against Gram-positive bacteria. Moreover, plasmid pNL1–03 carried the multi-copper oxidase gene *mco* and the copper-translocating P-type ATPase gene *copA*, which are involved in copper resistance. The second of the two UMCG578 plasmids, designated pUMCG578–02 with a size of 3,048 bp, was essentially identical to plasmid pRIVM1295–2 (query cover: 100%; identity: 99.88%) from the *S. aureus* strain RIVM1295 [5].

### Ethical statement

As the data presented in this article are results of the routine diagnostic investigations and patient details are not described, ethical approval was not needed.

## Discussion

The SCC*mec* elements precisely integrate into the *S. aureus* chromosome at a locus designated *attB*, located within the 3′ end of the *orfX* gene [6,7]. The Xpert MRSA NxG assay is a real-time PCR-based method designed to detect MRSA by targeting extremities of the chromosome–SCC*mec* junction (i.e. the *orfX* gene in *S. aureus* and the J3 region in SCC*mec*) [8]. This approach ensures a discrimination of MRSA from meticillin-susceptible *S. aureus* (MSSA) and meticillin-resistant coagulase-negative staphylococci if present together in a specimen. The Xpert MRSA NxG assay contains additional primers and probes that target a sequence in the *mecA*/*mecC* genes, reducing the possibility of a false-positive result [9]. The PCR primers designed for the Xpert MRSA NxG assay should efficiently hybridise to the targets in the NL1 and UMCG578 isolates. However, our investigation revealed that an integration of the SCC-like element at the 3’ end of the *orfX* gene resulted in the separation of *orfX* from SCC*mec* IVa, preventing amplification of the *orfX*-SCC*mec* target region.

Currently, there are two widely used commercial real-time PCR-based systems to detect MRSA directly from clinical samples, including the BD Max system (BD Diagnostics, Quebec, Canada) and Cepheid GeneXpert, described here. Both systems offer similar assays, which are based on targeting the 3’ end of the *orfX* gene in *S. aureus* and the J3 region in SCC*mec*. Recently, Monecke et al. reported false-negative test results in molecular MRSA identification using the Cepheid Xpert MRSA/SA BC and BD Max Staph SR assays [10]. In another study, Tenover et al. misclassified MRSA as MSSA based on the results produced by the Xpert MRSA/SA BC [11]. Both groups came to the same conclusion that the false-negative results produced by the assays likely resulted from the large insertions in the *orfX*/SCC*mec* integration site. Therefore, we can assume that the two MRSA ST398 isolates with the new SCC*mec*-CI characterised in our study may be misidentified in the *orfX*/SCC*mec* junction assays, which do not utilise a polymerase optimised for long-range PCR and increased extension time during target amplification.

Our analysis revealed several differences between the genomes of the NL1 and UMCG578 isolates. First, we only found the *Staphylococcus* prophage 96 in the NL1 isolate, while the StauST398–2 phage differed between isolates by several partial or entire gene deletions. Also, the isolates had different plasmid profiles, which underlie different antibiotic susceptibility patterns. Finally, the gene encoding a putative Ig domain-containing protein differed between isolates by a number of 267 nt repeats, and at least four transpositions of IS256 had occurred. These alterations suggest that the ST398 strain bearing the SCC-like element between *orfX* and SCC*mec* IVa has circulated for a longer time in the northern Netherlands, as it had already undergone microevolution. 

## Conclusion

We alert that this ‘false-negative’ MRSA strain could have already spread to other regions of the Netherlands and to other neighbouring countries. This could create a public health risk as this MRSA clone has been shown to have zoonotic potential, causing infections in people who come into contact with the carrier animals. Our study demonstrates that some MRSA ST398 strains may be missed using real-time PCR detection methods and highlights that molecular screening approaches should be performed in combination with culture-based identification and conventional antibiotic susceptibility testing.
